# Postural Control in Unipedal Quiet Stance in Young Female Gymnasts and the Effects of Training with Consideration of Transient Behavior of Postural Sway

**DOI:** 10.3390/ijerph19020982

**Published:** 2022-01-16

**Authors:** Urška Čeklić, Nejc Šarabon, Žiga Kozinc

**Affiliations:** 1 Faculty of Health Sciences, University of Primorska, Polje 42, SI-6310 Izola, Slovenia; urska.ceklic@fvz.upr.si (U.Č.); nejc.sarabon@fvz.upr.si (N.Š.); 2 Human Health Department, InnoRenew CoE, Livade 6, SI-6310 Izola, Slovenia; 3 Laboratory for Motor Control and Motor Behavior, S2P, Science to Practice, Ltd., Tehnološki Park 19, SI-1000 Ljubljana, Slovenia; 4 Andrej Marušič Institute, University of Primorska, Muzejski trg 2, SI-6000 Koper, Slovenia

**Keywords:** postural sway, balance, youth sport, stability, single-leg stance

## Abstract

The purpose of this study was twofold: (a) to compare postural control between a group of young female gymnasts (*n* = 15; age: 11.2 ± 1.9 years) and non-trained peers (*n* = 15; age: 10.9 ± 2.0 years), and (b) to investigate the effect of an 8-week whole body exercise intervention program on postural control in young female gymnasts. Postural control was assessed by recording center of pressure (CoP) movements during unipedal quiet stance. Velocity and amplitude of CoP movement in anterior-posterior (AP) and medial-lateral (ML) directions were considered. In addition to common trial-averaged CoP outcomes, we also considered the transient behavior of CoP movements, by calculating relative differences between the 1st and 2nd, and the 1st and 3rd 10-s intervals within the whole trial (DIF_21 and DIF_31, respectively). The gymnast group had lower total CoP velocity (Cohen’s d = 0.97) and AP amplitude (Cohen’s d = 0.85), compared to their non-trained peers. The gymnasts also had lower CoP AP amplitude DIF21 (Cohen’s d = 0.73), with almost constant values across all intervals. After the training ML CoP velocity was reduced for 13.12% (Cohen’s d = 0.60), while ML CoP amplitude increased (Cohen’s d = −0.89).

## 1. Introduction

Postural sway reflects the ability of individuals to maintain stable body position in static or dynamic situations. Postural control is developed and refined throughout childhood, and has an important influence on development of complex motor skills [1]. Postural control depends on sensory perception (visual, vestibular, proprioceptive) [2], sensory integration [3], and motor actions [4]. Gymnastics is a sport that involves very specific tasks, requiring high level of fine postural control [5,6]. Garcia et al. [7] suggested that performing activities with high demands for maintaining body orientation and equilibrium in gymnastics results in further refinement of postural control, compared to the general population. For instance, in gymnastics the athletes develop specific postural skills, such as a handstand and walking across a balance beam as quickly as possible [5,8]. Moreover, gymnasts often have to adopt unipedal rather than bipedal stance during their training [6], which results in improved postural control during specific, more complex gymnastic movements [9,10].

It is probably not surprising that several studies have indicated better postural control during unipedal tasks in gymnasts compared to non-gymnasts or other athletes [6,7,11,12]. On the other hand, some studies investigating postural control during bipedal standing have indicated no differences in the postural sway between gymnasts and other athletes, or gymnasts and non-trained peers [6,10,11,13]. This is in accordance with the observed task-specificity of balance ability, which suggests that training in certain balance tasks may have very little effect on performance of other balance tasks [14]. This was also observed specifically for gymnasts in a study that compared balance performance in bipedal, unipedal and handstand postures [5,15]. Based on the task-specificity principle, it seems reasonable to use unipedal tasks during training. However, the effect of additional supplementary training (i.e., performed in addition to regular gymnastic-specific training) for balance and strength training on unipedal balance performance in young gymnasts has not yet been explored. One study, conducted on university students specializing in gymnastics, explored the effect of a balance training program on postural stability [16]. They reported significant improvements in postural control in difficult tasks (i.e., with eyes closed or in unipedal stance), but not in easier tasks (i.e., bipedal tasks with eyes open).

One of the most common approaches to assess postural balance is by assessing postural sway via center-of-pressure (CoP) motion. In recent years, a new approach to analyze transient behavior of postural sway based on CoP data has been proposed [17]. Instead of averaging the CoP data across the trial, this approach separates the data into time intervals and assesses the changes in CoP behavior between these intervals. It was reported that indices of the transient behavior of postural sway are largely independent of the whole-trial estimates and were sensitive to age-related impairments in postural control, as well as to sensory conditions (vision elimination) [17]. Moreover, the sensitivity to leg preference [18], fatigue [19], and history of ballet dance training has also been shown [20]. Indices of transient behavior are purported to provide additional information regarding an individual’s balance control that are masked (e.g., initial less stable period) by averaging the data across the whole trial [17]. Given the nature of balance requirements in gymnastics, it is reasonable to expect that transient behavior indices of postural sway could be different in gymnasts compared to general population. Moreover, no study to date has examined the transient behavior of postural sway in children.

Therefore, the purpose of this study was to investigate the differences between young gymnasts and their non-trained peers in terms of postural sway during unipedal stance, including the traditional whole-trial outcomes, as well as the indices of transient behavior. The second aim was to explore the effects of supplementary exercise training in gymnasts on the same outcomes. We hypothesized that the gymnasts would exhibit better postural balance (lower CoP velocity and amplitude) and that they would stabilize more quickly than non-gymnasts (which will be reflected in different indices of transient behavior). According to recent evidence regarding the effects of fatigue and ballet dance training history [19,20], we hypothesized that the transient behavior indices, but not the whole-trial estimates of postural sway, would be affected by supplementary exercise training in gymnasts. We chose to study children and young adolescents, as postural control has been mostly investigated in youth and adult gymnasts so far. Gymnastics is a sport that requires an early specialization and high level of fine postural control, thus we expected to observe differences to the control group. At the same time, this group is probably more prone to improvements with training compared to youth and adult gymnasts with years of balance training experience.

## 2. Materials and Methods

### 2.1. Participants and Study Design

Our study included 15 female artistic gymnasts (11.19 ± 1.89 years, 143.63 ± 12.28 cm, 37.25 ± 10.34 kg, BMI: 17.62 ± 2.40, training years 3.4 ± 1.8, weekly training hours: 22 ± 6) and 15 similar-aged non-trained controls peers (10.92 ± 1.96 years, 146.71 ± 11.28 cm, 35.80 ± 11.03 kg, BMI: 16.36 ± 3.04). We used several inclusion criteria for the groups: (a) age between 9 to 15 years old, (b) no skeletal, muscle, nerve or connective tissue injuries during the last 12 months, (c) maximum of 2 h of physical activity per week for the control group. Additionally, to be included in the gymnasts’ group, the participants had to be involved in the training gymnastic club at least for 1 year. In the initial phase of the study, the two groups were tested (i.e., the gymnasts’ group that consisted of female gymnasts and the control group that consisted of non-active peers). A cross-sectional analysis was conducted to compare the two groups and infer the effects of being involved in gymnastic training. After the baseline measurements, the gymnasts’ group undertook a resistance exercise intervention program. After that, they came back for another measurement visit to assess the effect of training intervention. The experiment was approved by Republic of Slovenia National Medical Ethics Committee (approval number: 0120–690/2017/8) and was conducted in accordance with the Declaration of Helsinki.

### 2.2. Procedures

Before the measurements, the participants performed a warm-up that consisted of 6 min of walking on the stepper, stretching exercises for the main muscles’ group of the whole body and, at the end, 10 squats for the activation. After the warm-up, the participants were referred to one of the measurement stations. In this study, we included only static balance data (single-leg postural sway measurements), although we conducted several tasks in a random order: leg loading symmetry in standing and squatting position, postural sway, squat jump, countermovement jump, single-leg landing and maximal isometric strength assessments for lower limbs.

Postural sway measurements were conducted on a bilateral force plate (Kistler 3D, 9260AA, Winterthur, Switzerland). All participants were instructed to stand barefoot with eyes open, as still as possible for 30 s in unipedal posture. The opposite leg was flexed to 90° in the knee joint, and the participants were instructed to keep the hands on the hips. The gaze was oriented straight ahead to the target that was placed at their eye level on the wall, about 2 m away. Three trials were recorded in a random order for each participant (six trials in all). After each trial, participant rested for about 30 s and switched their leg. The data acquisition was started within ~1 s after the participants assumed the appropriate position.

### 2.3. Training Intervention

After the baseline measurements, the gymnast group went on to perform a two-part training intervention [21]. First, a whole-body resistance exercise program for five weeks, three times per week (session duration: 45 to 50 min). After the program was completed, the participants received additional special individualized training program for three weeks. This program was based on the detected inter-limb asymmetries in strength, flexibility and balance outcomes. Similar to the general program, three sessions of 45 min were carried out each week. The training involved tasks for improvement of: (a) balance (exercise consisting of single leg standing on unstable surfaces and single leg depth jumps), (b) range of motion and flexibility (hip flexors stretching with a partner, and stretching of hip external rotators), and (c) resistance exercises (consisting of back plank with single leg lift and single leg slides, single leg step-ups and jumps on the box, as well as side plank on the floor). Training volume and intensity of all the tasks were progressively increased during the three week period. Participants performed 3–4 sets of each task, and the breaks between repetitions were set to 2–3 min.

### 2.4. Data Processing and Outcome Measures

The ground reaction force data were sampled (acquisition frequency: 1 kHz) with a force plate (type 9260AA; Kistler, Winterthur, Switzerland). The data were low-pass filtered with a 2nd order, Butterworth filter at 10 Hz. This was done automatically within the manufacturer’s software (MARS, version 4.0; Kistler, Winterthur, Switzerland). The data were further automatically processed in the same software to obtain the following outcome variables: mean CoP velocity (total, anterior–posterior (AP), and medial–lateral (ML)) and CoP amplitude (AP and ML). These two variables were chosen because they showed the highest reliability in our pilot trials. The CoP amplitude was defined as the average amount of the CoP sway in AP or ML direction, calculated as the common length of the trajectory of the COP sway only in the given direction, divided by the number of changes of movement direction. Firstly, we calculated the traditional whole-trial estimates (i.e., averaged CoP characteristics, as defined above, over the whole 30 s of the trial). Then, we also calculated the quotients between the 1st and the 2nd (DIF_21) and the 1st and the 3rd (DIF_31) 10 s time intervals within the whole trial, which were expressed as percentages (100% representing no change, >100% indicating an increase in time, <100% indicating a decrease in time) [18,19]. For all the outcome variables, we used the mean value of the three repetitions.

### 2.5. Statistical Analysis

Statistical analyses were done with SPSS (version 25.0, SPSS Inc., Chicago, IL, USA). Descriptive statistics are reported as mean ± standard deviation. The normality of the data distribution was checked with Shapiro–Wilk tests. Correlations among whole-trial variables and transient behavior indices were assessed with Pearson’s correlation coefficients and interpreted as negligible (<0.1), weak (0.1–0.4), moderate (0.4–0.7), strong (0.7–0.9) and very strong (>0.9) [22]. The differences between the groups were assessed with independent-samples *t*-test, and the effects of training with pair-wise *t*-tests. Hedges’ g effect sizes (ES) were calculated as suggested by Rhea where: <0.25 = trivial; 0.25–0.50 = small; 0.51–1.0 = moderate and >1.0 = large [23]. The threshold for statistical significance was set at *p* < 0.05.

## 3. Results

### 3.1. Correlations among Whole-Trial Variables and Transient Behavior Indices

There were two statistically significant small to moderate correlations between whole-trial estimates and the DIF-31 of the corresponding variable (CoP velocity total and CoP ML velocity; *r* = 0.43 and 0.36, respectively). Additional small to moderate correlations were present across whole-trial estimates and non-corresponding transient behavior indices (see Table 1 for details). Note that these results refer to correlations performed on a whole study sample (gymnasts and non-gymnasts). Group-specific correlations were even smaller and exclusively statistically non-significant (*p* ≥ 0.138).

### 3.2. Comparison of Gymnasts and Control Groups 

Table 2 displays descriptive statistics for the gymnasts’ group (baseline values) and non-gymnast groups. The gymnasts’ group had moderately lower total CoP velocity (45.09 ± 0.76 vs. 56.82 ± 15.36 mm/s; *p* = 0.33; ES = 0.97) and moderately lower CoP AP amplitude (5.27 ± 1.41 vs. 6.63 ± 1.78 mm; *p* = 0.036; ES = 0.85). All of the remaining whole-trial variables showed similar trends and moderate ES, but the differences between the groups were not statistically significant. The gymnasts also had lower indices of transient behavior across all variables (ES = 0.08–0.73), but the differences between the groups were statistically significant only for CoP AP amplitude DIF21 (*p* = 0.040; ES = 0.73). The values of DIF21 imply that the gymnasts’ postural sway slightly decreased from 1st to 2nd interval (DIF21 = 97.49 ± 9.73), whereas it increased in the non-gymnast group (DIF 21 = 113.25 ± 29.05).

### 3.3. Effects of Training

Table 3 shows the descriptive statistics of baseline and post-training outcomes. ML CoP velocity was reduced for 13.12%, which was reflected in moderate effect size (*p* = 0.041; ES = 0.60). On the contrary, the ML CoP amplitude increased, which was reflected in large effect size (*p* = 0.002; ES = −0.89). No other statistically significant differences were observed.

## 4. Discussion

The first aim of the present study was to investigate the differences between young gymnasts and their peers in terms of postural sway during unipedal stance, including the traditional whole-trial outcomes, as well as the indices of transient behavior. In terms of the whole-trial estimates, statistically significant differences were shown between the groups regarding the CoP total velocity and CoP AP amplitude, with the gymnasts exhibiting superior balance ability (see Table 2 for details). Moreover, the index of transient behavior (DIF-21) for CoP AP was also pointing towards differences between the groups. The second aim was to assess the effects of training intervention in gymnasts. We found that the CoP velocity decreased and CoP amplitude increased after the training, while there were no changes in indices of transient behavior.

Our results suggest that balance ability is improved as a result of gymnastic training in youth. In contrast, a study of Opala–Berdzik et al. [13], conducted on gymnasts and a non-active control group, using bipedal quiet stance, showed no statistically significant differences in CoP velocity between groups. Gymnasts are largely trained to maintain a unipedal posture or non-parallel bipedal posture. Thus, it seems reasonable to expect that differences compared to control populations are evident in unipedal, but not bipedal-parallel stance tests [5,11,15]. Namely, the adaptations to balance training have been reported to be highly task-specific [14]. To the best of our knowledge, no study that evaluated postural sway using unipedal stance has compared female gymnasts and non-gymnasts. Asseman et al. [11] assessed balance in male gymnasts and other athletes using unipedal stance. They found that gymnasts had significantly better balance than other sportsmen. It seems that differences between groups are indeed detected more easily in unipedal than bipedal stance, so unipedal stance should be used to investigate differences in the future. Our results showed group differences in the index of transient behavior in CoP AP-DIF21, which was lower in gymnasts compared to control peers. This is in accordance with our previous study conducted on ballet dancers and healthy adults [20]. Similar to our results, that study suggested that transient postural sway characteristics could provide additional information regarding the postural control in the athletes, and that the transition to single-leg stance could be one of the maneuvers causing the initial destabilization period. The results related to the transient body sway characteristics in our study indicated that the gymnasts maintained similar CoP behavior throughout the trial (see Table 2).

Gymnastics requires different postures and balance exercises that are often implemented in the training process with the aim of optimizing performance, preventing injury or providing rehabilitation [24,25]. The effects of training in gymnastics on postural performance and postural control have not yet been investigated. The results of our intervention did not indicate effects of intervention training on postural control during unipedal stance. We found a statistically significant effect on CoP VEL ML (*p* = 0.041), which decreased in post intervention measurement, meanwhile CoP AMP ML (*p* = 0.002) increased after the intervention. Other parameters of CoP did not show effects of the intervention. The explanation for lack of changes in several outcomes can be due to the fact that our group of gymnasts were already included in the training process for a few years. Therefore, probably the effect of additional postural training did not noticeably influence the participants’ postural control. On the contrary, studies conducted on different athlete groups demonstrated improvements in CoP using unipedal stance after the balance intervention [26,27]. On the other hand, a study conducted on a recreational population did not report statistically significant improvements in postural control in ML and AP directions [28]. Nonetheless, no studies to date have examined the effects of intervention on postural sway in gymnasts. An unexpected result was a rather high increase in CoP ML amplitude after training. One explanation could be that the participants learned (with training) to be more stable and confident over a larger portion of the base of support. Studies on older adults and patient populations have suggested that larger postural sway might not always suggest poor balance control and vice versa [29].

The transient body sway characteristics have been suggested as potentially relevant for clinical assessment of risk of falls [20], and have been shown to be sensitive to age [17]. We hypothesized that transient body sway characteristics might be more sensitive to the effect of training, but we did not confirm this in the present study. The reason could be in relatively short-term period of training on young sportsmen, who were already included in the training process before the study. We confirmed that the indices of transient behavior are mostly independent of the whole-trial postural sway variables (as shown by absent or low correlations). This is important, as it suggest that there is a potential independent information regarding postural control that is obtainable with indices of transient behavior.

We would like to highlight some limitations of our study. The intervention part of the study did not include a control group, which limits the interpretation of the results. Moreover, some studies used eyes closed conditions in gymnasts to assess balance, which could be more sensitive to changes induced by training. The sample size of the study was relatively low, which was due to the inaccessibility of appropriate participants. Note that many of the previous studies that investigated postural control in young and adult gymnasts investigated had similar sample sizes [5,6,7,11,13]. Moreover, the sample size was limited to young female gymnasts. The results should not be generalized across gymnasts of different age groups and to male gymnasts. Future studies are needed and should use larger samples, comprised of both male and female gymnasts, and use of control groups.

## 5. Conclusions

This study showed that differences in postural control exist between young female gymnasts and non-gymnast peers. In addition to exhibiting lower postural sway, gymnasts also showed a more constant CoP behavior throughout the trial, which was reflected in different indices of transient behavior between the groups. The effect of training on transient behavior of postural sway was not observed, whereas the results regarding whole-trial variables were ambiguous. It is suggested that supplementary training in youth female gymnasts elicits little to no improvements in balance and postural control compared to gymnastic training alone.

## Figures and Tables

**Table 1:** The correlations between whole-trial variables and indices of transient behavior of body sway.

Outcome Variable	CoP VEL Total	CoP VEL AP	CoP VEL ML	CoP AMP AP	CoP AMP ML
CoP VEL—DIF21	0.05	−0.03	0.06	0.05	0.00
CoP VEL—DIF31	0.43 *	0.10	0.46 *	0.04	0.24
CoP VEL AP—DIF21	0.11	0.14	0.06	0.15	−0.03
CoP VEL AP—DIF31	0.39 *	0.25	0.36 *	0.20	0.13
CoP VEL ML—DIF21	0.08	−0.34	0.23	−0.17	0.10
CoP VEL ML—DIF31	0.30	−0.01	0.36*	−0.09	0.18
CoP AMP AP—DIF21	0.37 *	0.30	0.31	0.24	0.14
CoP AMP AP—DIF31	0.54 **	0.37 *	0.49 **	0.27	0.28
CoP VEL ML—DIF21	−0.05	−0.43 *	0.13	−0.21	−0.01
CoP VEL ML—DIF31	0.16	−0.15	0.25	−0.19	0.06

CoP—center of pressure; VEL—velocity; AMP—amplitude; AP—anterior-posterior; ML—medial-lateral; * *p* < 0.05; ** *p* < 0.01.

**Table 2:** The comparison between gymnasts (*n* = 15) and non-gymnast groups (*n* = 15).

Outcome Variable	Gymnasts		Non-Gymnasts		Difference	
	Mean	SD	Mean	SD	*p*	ES
CoP VEL total (mm/s)	45.09	7.68	56.82	15.36	0.033	0.97
CoP VEL AP (mm/s)	27.61	6.27	31.46	6.38	0.110	0.61
CoP VEL ML (mm/s)	29.77	5.97	39.59	16.43	0.101	0.79
CoP AMP AP (mm/s)	5.27	1.41	6.63	1.78	0.036	0.85
CoP AMP ML (mm/s)	2.98	1.11	4.13	1.94	0.085	0.73
CoP VEL—DIF21	95.34	5.09	102.34	16.20	0.097	0.58
CoP VEL—DIF31	95.02	9.27	98.39	13.24	0.310	0.30
CoP VEL AP—DIF21	97.25	5.55	106.58	21.14	0.310	0.60
CoP VEL AP—DIF31	96.85	8.40	101.69	18.20	0.548	0.34
CoP VEL ML—DIF21	93.34	7.06	97.80	9.41	0.101	0.54
CoP VEL ML—DIF31	94.34	19.14	94.94	11.78	0.310	0.04
CoP AMP AP—DIF21	97.49	9.73	113.25	29.05	0.040	0.73
CoP AMP AP—DIF31	95.75	13.13	105.88	30.28	0.419	0.43
CoP AMP ML—DIF21	90.43	14.27	97.65	18.30	0.191	0.44
CoP AMP ML—DIF31	91.55	29.49	89.66	16.83	0.494	0.08

CoP—center of pressure; VEL—velocity; AMP—amplitude; AP—anterior-posterior; ML—medial-lateral; ES—effect size (Hedges’ g).

**Table 3:** The comparison of baseline and post-training results (*n* = 12).

Outcome Variable	Baseline		Post-Training		Difference	
	Mean	SD	Mean	SD	*p*	ES
CoP VEL total (mm/s)	44.60	8.12	39.92	9.93	0.084	0.52
CoP VEL AP (mm/s)	27.32	6.72	25.67	6.71	0.638	0.25
CoP VEL ML (mm/s)	29.44	6.57	25.58	6.22	0.041	0.60
CoP AMP AP (mm/s)	5.05	1.49	4.83	1.59	0.530	0.14
CoP AMP ML (mm/s)	3.65	1.03	4.92	1.73	0.006	−0.89
CoP VEL—DIF21	95.46	5.63	94.83	8.90	0.695	0.08
CoP VEL—DIF31	93.80	7.99	92.42	10.58	0.480	0.15
CoP VEL AP—DIF21	98.20	5.74	95.67	10.41	0.308	0.30
CoP VEL AP—DIF31	97.71	9.12	95.42	15.84	0.583	0.18
CoP VEL ML—DIF21	92.74	7.67	94.50	8.68	0.583	−0.21
CoP VEL ML—DIF31	89.65	9.71	90.25	7.88	0.754	−0.07
CoP AMP AP—DIF21	99.23	10.14	98.00	15.92	0.530	0.09
CoP AMP AP—DIF31	97.58	13.76	100.75	29.29	0.695	−0.14
CoP AMP ML—DIF21	90.19	15.82	91.25	16.99	0.875	−0.06
CoP AMP ML—DIF31	85.74	22.00	95.25	17.49	0.158	−0.48

CoP—center of pressure; VEL—velocity; AMP—amplitude; AP—anterior-posterior; ML—medial-lateral; ES—effect size (Hedges’ g).

## Data Availability

Not applicable.
